# Sensing of Magnetic-Field Gradients with Nanodiamonds on Optical Glass-Fiber Facets

**DOI:** 10.1021/acsanm.3c00887

**Published:** 2023-06-14

**Authors:** Mona Jani, Paulina Czarnecka, Zuzanna Orzechowska, Mariusz Mrózek, Wojciech Gawlik, Adam M. Wojciechowski

**Affiliations:** Marian Smoluchowski Institute of Physics, Jagiellonian University, Łojasiewicza, 11, 30-348 Kraków, Poland

**Keywords:** nanodiamond, photonic sensor, ODMR, nitrogen-vacancy, fluorescence, magnetic field gradients

## Abstract

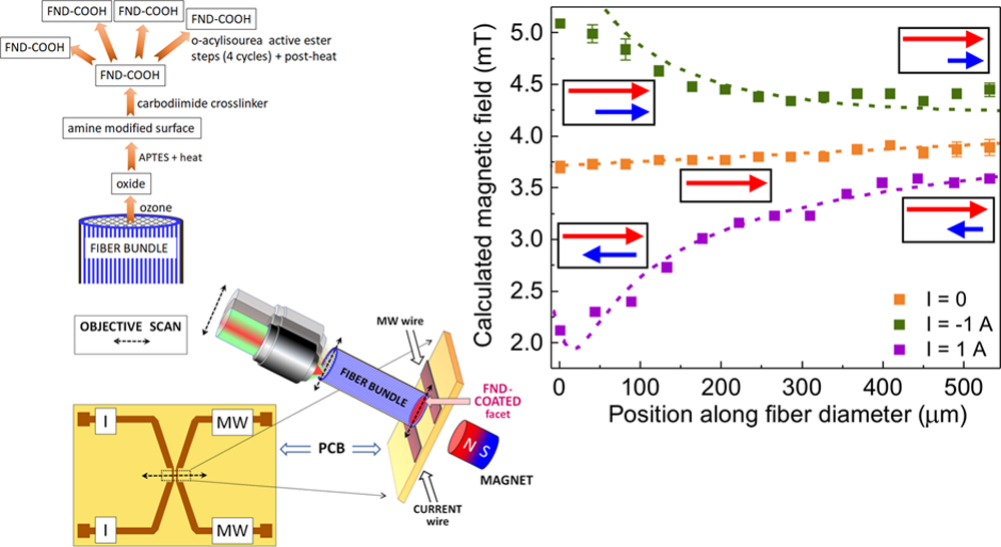

We demonstrate a photonic sensor of the magnetic field and its gradients with remote readout. The sensor is based on optically detected magnetic resonance (ODMR) in nanodiamonds with nitrogen-vacancy color centers that are covalently attached as a thin film on one facet of an optical fiber bundle. By measuring ODMR signals from a group of individual fibers in an ∼0.5-mm-wide imaging bundle, differences of local magnetic field strengths and magnetic field gradients are determined across the plane of the bundle facet. The measured gradients are created by direct electric currents flowing in a wire placed near the nanodiamond film. The measurement enabled the determination of the net magnetic field corresponding to various current directions and their corresponding magnetic field gradients. This demonstration opens up a perspective for compact fiber-based endoscopy, with additional avenues for remote and sensitive magnetic field detection with submicrometer spatial resolution under ambient conditions.

## 1 Introduction

Integration of fluorescent nanodiamonds (FNDs) containing negatively charged nitrogen-vacancy (NV) centers with optical glass fibers is a crucial area of research for remote nanoscale magnetometry^1−4^ and thermometry.^5,6^ Electronic spin states of NVs can be manipulated using microwave (MW) fields, and the change of their state can be read out using the optically detected magnetic resonance technique. As NV centers are sensitive to their environment, FNDs have been used to measure parameters such as local magnetic field,^7^ temperature,^8^ and concentration of: spin labels,^9^ ions in solution,^10^ and free radicals (iron-containing proteins).^11^ They have also been useful for the development of relaxometry methods, e.g., ref (11). The use of optical fibers allows the development of a compact endoscopy approach, where NV centers can be excited and their emission can be collected along the same fiber from remote distances.^2,4,12^ Integration of FND with fibers has been demonstrated by coupling FNDs with single-mode fibers,^13^ photonic crystal fibers,^14^ and fiber tapers.^15^ To date, detection of local magnetic fields by side excitation and longitudinal collection has been performed using the ODMR technique with FND and microdiamonds doped in tellurite,^2,16^ and lead silicate glass fibers.^17^ In addition, an MW-free detection mode was demonstrated in ref (12) where it was shown with submicrometer diamond particles embedded in 60 cm long lead silicate glass fiber that the fluorescence level can be related to the magnetic field value in a broad dynamic range of 0–35 mT. Furthermore, a multichannel fiber sensor capable of parallel-in-time mapping and instantaneous readout of individual pixels was demonstrated and allowed magnetic mapping with high spatial resolution using the FND-coated fiber facet.^4^ Despite these efforts, FND-fiber sensors have not yet reached the level of traditional optical platforms, and further development is needed to achieve sensitive measurements of magnetic field distributions with high spatial resolution and real-time readout over remote distances.

Here, we present our attempt to solve this task with a surface-chemical approach by covalently anchoring carboxylated FNDs in a thin film on a facet of a glass imaging fiber bundle (IFB) that is surface-functionalized with (3-aminopropyl)triethoxysilane (APTES), following Patoary et al. who prepared the nanodiamond thin films on plain-glass substrate.^18^ With this approach, we managed to procure robust FND thin films with high interface quality. NV fluorescence from the appropriately coated IFB front facet and its propagation through the IFB were detected using wide-field microscopy and confocal microscopy. Variations of the magnetic field strength occurring at one facet of the FND-anchored fiber are simultaneously read as the fluorescence variation through the other end facet of the same fiber. In our previous article,^4^ we reported observation of ODMR signals from FNDs through single cores of IFB, albeit with a low signal-to-noise ratio, which hindered practical magnetic field measurements. In a related work,^19^ it was observed that the ODMR signals became very noisy due to background fluorescence when the region of interest decreased. To mitigate such a problem, in this work, we applied an improved camera system and imaging protocol, which have resulted in great enhancement in the signal-to-noise ratio of the recorded ODMR signals, thus enabling precise detection of magnetic field gradients.

In the following, we describe an application of an IFB appropriately coated with FND for monitoring magnetic field variations. This proof-of-principle experiment demonstrates sensitive mapping with a high spatial resolution of the magnetic fields around a micrometer-thin current-conducting wire near the FND film. We hope that it enables a significant step toward the development of nanosensing technology by remote and instantaneous mapping of the magnetic field gradients under ambient conditions.

## 2 Experimental Section

### 2.1 Chemical Thin-Film Assembly of FNDs on the Facet of a Fiber Bundle

Conventional approaches for the integration of FNDs in optical fibers include embedding FNDs in molten glass during the direct fiber fabrication process,^2,12,20,21^ attaching them by fusing,^22^ gluing,^23^ or depositing on the fiber facet.^24,25^ However, these standard methods limit the selection of applicable fiber types, hinder optical readout sensitivities, or are overly complex. An alternative approach, which we applied in this study, is the use of a silanization method, to covalently attach FNDs to the chosen glass fiber. We followed a deposition protocol laid out in ref (18), where the authors describe in detail the formation of a covalently bonded nanodiamond network, its spectroscopic characterizations, and surface morphology on glass substrates. We were able to form uniform and thin layers of FND having the Volmer–Weber type of growth over large surface areas on glass substrates and on fibers with very specific glass compositions.^4^ The silanization method used for covalently assembling FNDs on glass is sensitive to parameters such as concentrations, solvent quality, temperature, and reaction times. Variations in these parameters can negatively impact the adhesion of the film. A high-density silanization of the glass surface with APTES is crucial in the creation of the thin film because it determines the efficiency of the covalent bonding of the FNDs with the surface-terminating amino groups. APTES is currently one of the most widely used organosilane agents for the preparation of amino-functionalized organic thin films,^26^ for the chemical incorporation of nanoparticles,^27^ proteins,^28^ and DNA^26^ in substrates. Several studies suggested that heat treatments densify the modified APTES layers by horizontal heat-enhanced polymerization,^29,30^ reduce the thickness of an APTES film, and crosslink silane molecules on the surface by eliminating H_2_O molecules, forming a more robust silane layer.^31^ Curing functionalized APTES substrates between 70 and 150 °C eliminates a substantial amount of loosely bound APTES containing protonated amino groups from the surface by evaporation or condensation.^29^ Moreover, most ethoxy groups are removed, and the surface is covered with a new class of well-ordered neutral amino groups that are more reactive than the protonated buried and/or randomly oriented amino groups of the film before heat treatment. Furthermore, the amino-functionalized layer is more hydrolytically stable in an aqueous solution than the layers prior to curing and exhibits a strong antigen-binding capacity.^32^

We modified our previous deposition protocol,^4^ which lacked heat treatments, by incorporating intermediate heating steps in the procedure to obtain an APTES monolayer coating at the glass interface that is chemically, mechanically, and hydrolytically stable in an aqueous solution with a high number of accessible reactive amino groups well ordered at the interface. The developed procedure resulted in a dense coverage with good homogeneity of the deposited thin films of FND on the optical glass-fiber facet (shown in Figure 3b in Section 3.1) relative to the simple drop-drying deposition of FNDs. Furthermore, whereas previously, the FNDs uniformly assembled on APTES-functionalized glass surfaces were very prone to detach; we noticed that heat treatments did not remove the physisorbed molecules, but enhanced the crosslinking reaction of the silane layer, thus improving the bond stability at the glass interface.^29,33^

A suspension of carboxylated FNDs (FND-COOH) with a size of 140 nm and 1.5 ppm concentration of NV centers was obtained from Adamas Nanotechnologies. The spectroscopic properties of these FNDs have been previously characterized in ref (34). The imaging fiber bundle (IFB) had cladding made of soda-lime-silicate glass (SK222) with a refractive index of *n*_d_ = 1.522 and the cores were made of barium–zirconia–borosilicate glass (Zr3/XV) with *n*_d_ = 1.611.^35,36^ Individual pixels in the bundle had a diameter of 2.8 μm, while the entire bundle had core and clad diameters of 0.532 and 0.550 mm, respectively.

In our approach, IFB was first hydroxylated with ozone for 30 min (Ossila UV Ozone Cleaner) and then immediately immersed in a 0.01% (v/v) solution of APTES in toluene for 30 min. This resulted in covalent interactions between the hydroxyl groups present on the surface and the hydrolyzed silane molecules *via* hydrogen bonds. One end of IFB facet was then sonicated in toluene for 5 min to remove any physisorbed APTES molecules and heated at 150 °C for 10 min on a heating plate to evaporate excess molecules that were not bound to the surface. The procedure resulted in a well-anchored and stable cross-linked layer of APTES.

**Figure 1:**
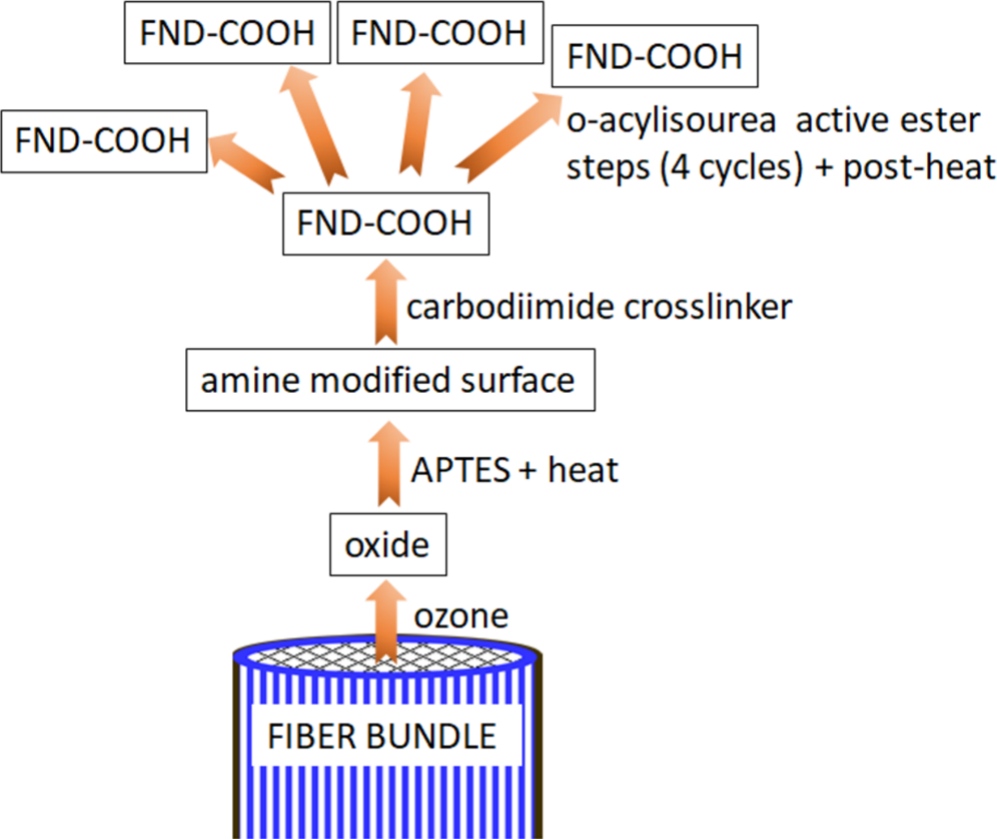
Schematic diagram to show steps followed to covalently anchoring carboxylated FNDs in a thin film on a facet of a glass imaging fiber bundle (IFB) that is surface-functionalized with (3-aminopropyl)triethoxysilane (APTES).

Separately, an amine-reactive *O*-acylisourea solution of FND-COOH was prepared by mixing 1 mg/mL FND-COOH in a 1:1 ratio (v/v) with 0.3 mg/mL 1-ethyl-3-(3-dimethylaminopropyl)carbodiimide hydrochloride in a buffer solution of 2 mM potassium chloride (pH 6.5). The APTES-functionalized IFB facet was then immersed in this solution for 30 min to complete the first cycle of formation of a thin layer of FNDs on the glass surface. Four additional cycles of regeneration were carried out using a cyclic procedure involving ethylenediamine followed by amine-reactive *O*-acylisourea steps to increase the density of the FND thin-film layers. The resulting covalently thin-film assembled FND on the IFB facet was then rinsed with deionized water, naturally dried, and post-heated at 150 °C for 15 min. Post-heating provided a great improvement in the anchoring and bond strength of the FND film on glass surfaces, including its robustness and quality. The FND thin film was stable in general handling throughout our experiments and, in particular, remained less scratched compared to the physically deposited FNDs in our previous attempts.^37^ Finally, we washed the formed FND film with deionized water and dried it in argon. In Figure 1, we illustrate schematically the applied chemical technology. As not all diamonds had equal sizes and shapes,^38^ FNDs were not uniformly attached to the silanized surface, and therefore it was not easy to obtain quantitative information on the attachment strength of FNDs. However, we could observe that the thin film of FNDs attached on the IFB was stable and resistant to scratches. In particular, it worked efficiently for more than 5 months despite frequent contacts with the antenna board.

### 2.2 Fluorescence Microscopy and ODMR Setups

In our work, two imaging methods have been used: wide-field microscopy and confocal fluorescence microscopy. They are based on two separate but complementary setups that work in the epifluorescence mode. Although the confocal fluorescence setup has a high resolution and a different technique for excitation than the wide-field technique, the latter is very suitable for imaging large areas.

The wide-field fluorescence microscopy images were acquired using a Motic BA210E microscope operating in the epifluorescence mode. A green LED light source was used to excite the FNDs assembled on the fiber facet, and the images were taken with 40×/0.65 (LUCPLFLN, Olympus) objective captured with a digital camera (Moticam3+).

The confocal images were acquired using a home-built microscopy setup with an oil-immersion objective (100×/1.3, UPLFLN Olympus) and a 532 nm laser (Sprout G, Lighthouse Photonics, attenuated to ∼1 mW). After laser excitation of FNDs, their backward-emitted fluorescence was separated by a dichroic mirror and filtered spatially and spectrally (FELH600, Thorlabs) before being detected by a single-photon counting module (SPCM-AQRH-14-FC, Excelitas Technologies) and the images were collected by scanning the sample above the objective using a piezo-nanopositioning stage (Nano-LP200, Mad City Labs) controlled by a PC with Qudi software.^39^

**Figure 2:**
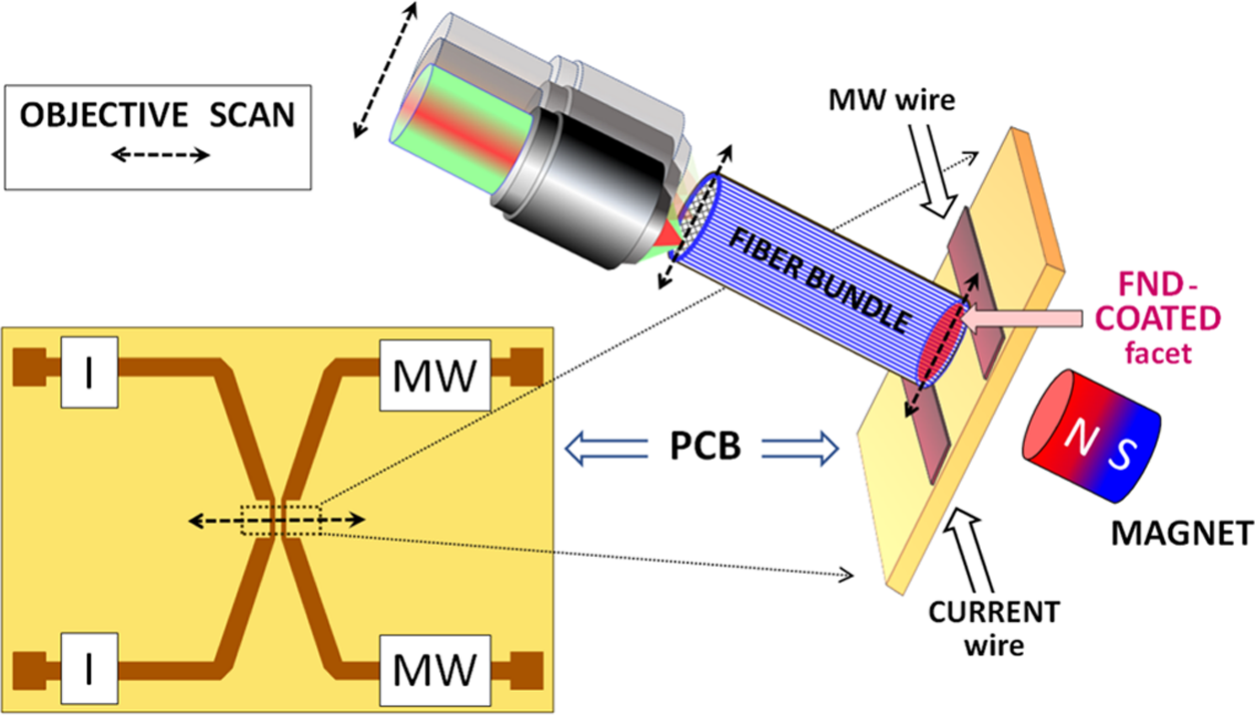
Schematics of the ODMR setup showing the fiber bundle (IFB) with its coated and uncoated facets, the microscope objective, the permanent magnet creating the bias field, and the antenna with two striplines: the MW and current (*I*) wire. The laser light illuminates the uncoated facet and excites NV centers in FNDs placed on the coated facet. The emitted NV fluorescence, which depends on the local MW and magnetic field strengths, is collected through the fiber bundle and transmitted backward by IFB toward the objective to be detected and yields the ODMR signal. Dashed double-ended arrows indicate the scan direction of: the moving objective, the laser beam focused on the uncoated IFB facet, and the scanned laser beam over the specific spot on the coated IFB surface between the striplines.

The arrangement to collect the ODMR signals through IFB comprises a home-built, wide-field microscopy setup and is shown schematically in Figure 2. Approximately 65 mW of the green laser power was focused on the back-focal plane of the 40×/0.6 objective (LUCPLFLN, Olympus) and transmitted through IFB from its uncoated facet to the one coated with FND. The coated facet of IFB was centered between two parallel copper striplines (34 μm-high, 170 μm-wide, and 2.4 mm-long) on a printed circuit board, for brevity called the “antenna”,^40^ and was placed 0.2 mm above the antenna. The NV fluorescence excited in FND was transmitted backward through IFB and the objective and was detected by a camera.

One of the antenna striplines transmitted the MW signal to manipulate the ground-level spin states of the NV centers. Another stripline, separated by 320 μm, conducted a direct electric current from a stable current source to create a given spatial magnetic field distribution. An additional and stronger bias field of *B*_0_ = 3.8 mT was applied using a permanent magnet to lift the degeneracy of the *m*_s_ = ±1 states and had a normal direction to the antenna board. The continuous-wave MW field was generated by a signal generator (SG386, Stanford Research Systems), and its frequency was swept around the resonance frequency of the *m*_s_ = 0 – *m*_s_ = ±1 transition at 2870 MHz with steps of 0.5 MHz. The red fluorescence (ca. 600–800 nm) transmitted through the fiber bundle was captured by the microscope objective, filtered, and projected onto the sCMOS camera (Andor Zyla 5.5). ODMR data collection was performed in LabView software, which controlled the camera, signal generator, and digital pattern generator (Pulse Streamer 8/2, Swabian Instruments). During continuous light illumination, two images were captured with MWs turned on and off for every frequency to subtract the background and calculate the normalized fluorescence contrast, which helped to reduce fluorescence intensity variations due to laser power drifts and improved the overall signal-to-noise ratio.

Images given by the central 704 × 594 pixels were acquired, corresponding to a full frame (FF) field of view of 205 μm × 173 μm and an effective pixel size of ∼(0.55 μm)^2^. To achieve a high spatial resolution while maintaining a sufficient signal-to-noise ratio, fluorescence from smaller areas of interest (AOIs) was acquired. Each AOI had dimensions of ∼15.6 μm × 15.6 μm, equivalent to 22 individual fibers in the IFB. The fluorescence collected within each AOI was integrated and ascribed to a given position *r* between the antenna wires. Figure S1 illustrates the selection of individual AOIs and their application to map of the magnetic field distribution near the current-conducting wire along the entire width of the IFB. The dependence of integrated fluorescence intensity on the distance *r* from the current wire enabled the determination of the spatial distribution of the magnetic field.

## 3 Results and Discussion

### 3.1 Imaging Fluorescence of an FND Film

**Figure 3:**
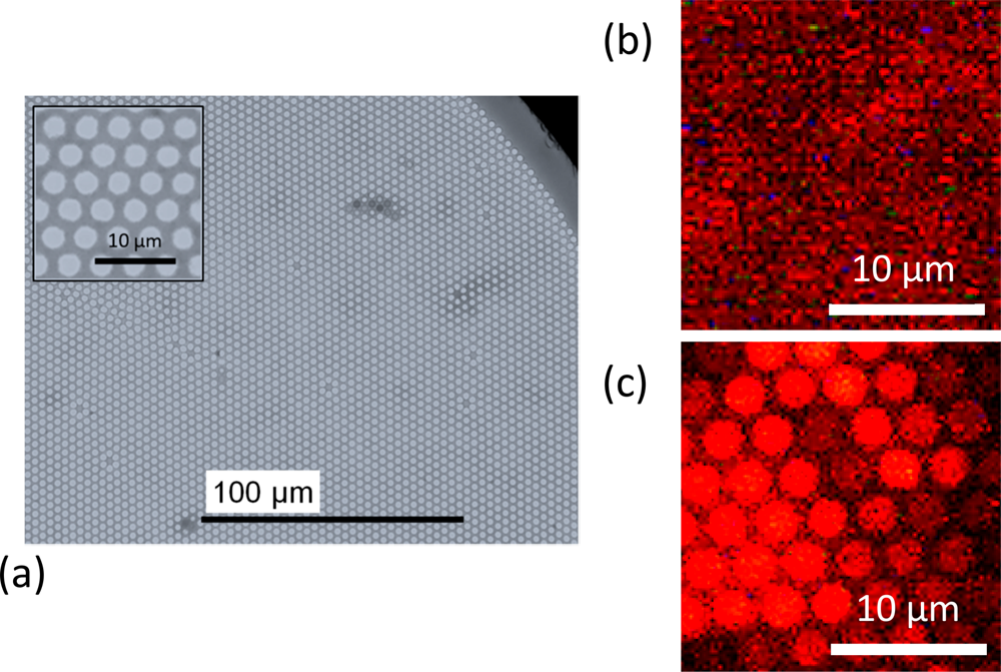
Wide-field microscope images of IFB: (a) fiber structure with individual pixels of 2.8 μm diameter; (b) red fluorescence of the IFB facet coated with FND excited by green light; (c) red fluorescence of FNDs seen from the uncoated side of IFB, excited by green light.

As described in Section 2.1, to achieve sufficiently dense and uniform films with covalent assemblies of FNDs on an end facet of IFB, four coating cycles followed by thermal treatments were performed. Figure 3a shows the wide-field microscope image of the clean IFB facet before FND deposition. Individual fibers of the IFB with 2.8 μm diameter are easily visible as bright spots due to a relatively high-refractive-index contrast between the fiber core and the fiber cladding that eliminates optical crosstalk between individual fibers. The IFB had a sufficiently high numerical aperture (NA > 0.5) to allow efficient observation of the fluorescence emitted from its coated facet and allow its transmission throughout the bundle. Figure 3b,c presents fluorescence images from an epifluorescence microscope that displays the coated and the uncoated facets of the IFB, respectively. In Figure 3b, the red fluorescence is emitted by the NV centers deposited on the coated IFB surface and shows that the FND film obtained is relatively homogeneous and exhibits a high degree of coverage. When the IFB ends were interchanged to observe the uncoated side, as shown in Figure 3c, each core appeared much brighter than the cladding, due to efficient fluorescence guidance in the individual IFB cores.

**Figure 4:**
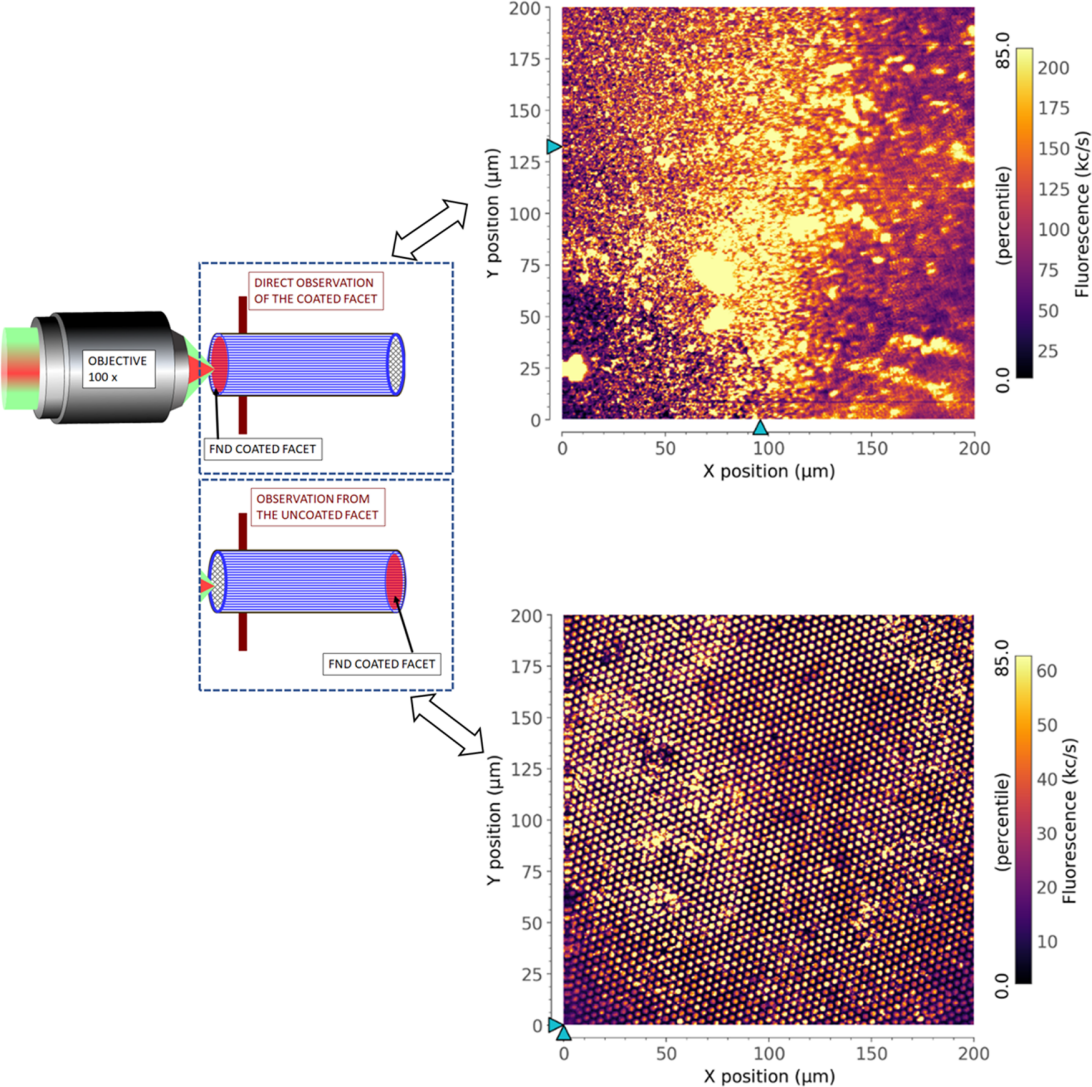
Schematics of confocal microscopy observation of both facets of IFB (left) and confocal maps showing fluorescence from the FND film obtained with four regeneration cycles: the top image directly shows the coated facet, and the lower image shows fluorescence recorded through the IFB, i.e., from the uncoated side.

To confirm the excitation and distribution of FNDs on the coated IFB facet and efficient propagation of NV fluorescence through the IFB, we have used scanning confocal fluorescence microscopy as schematized in Figure 4 (left). Typical images with confocal fluorescence intensity maps of the 200 μm × 200 μm region are shown in the top image for the FND-coated IFB facet and in the lower image for the uncoated side. The direct NV emission from the coated facet appears with a higher fluorescence photon count rate of about 250 kc/s, while collecting photons through the other end resulted in lower photon counts of about 65 kc/s. Confocal scanning maps confirm that FNDs are uniformly distributed over the entire fiber facet and show that the NV emission efficiently couples to the guided optical modes of the IFB.

### 3.2 Detection of Magnetic Field Gradients through the IFB

#### 3.2.1 ODMR Spectra

**Figure 5:**
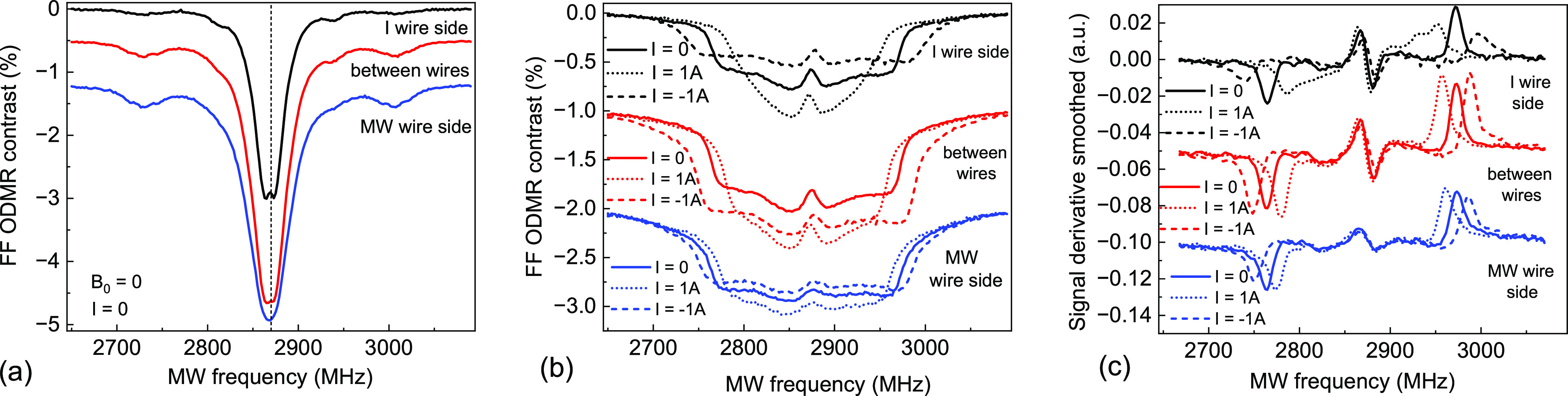
ODMR signals collected through the IFB at different positions between antenna wires along the fiber diameter [direction indicated in Figure 2 by the double-arrowed dashed line]. (a) Zero magnetic field, zero current. At *B*_0_ = 3.8 mT for different current intensities: (b) regular ODMR signals and (c) derivatives of ODMR signals. The outer peaks in (b, c) correspond to the edges of the ODMR spectrum and clearly shift with magnetic field changes. Only the top curves correspond to the left scales, the other curves are vertically offset to avoid their overlap.

NV magnetometry is a very successful method of studying bulk diamonds. As shown in our earlier work,^37^ ODMR can also be very useful with ensembles of arbitrary-oriented FNDs with NV. When there is no magnetic field, the ODMR spectrum exhibits a standard resonance centered at 2870 MHz, as illustrated in Figure 5a. When a sufficiently strong magnetic field is applied, the ODMR resonance in FNDs broadens and loses its contrast, due to averaging over arbitrary orientations of the projections of NV axes of individual particles. Importantly, this broadening is approximately proportional to the magnetic field strength and enables its determination. However, to observe and quantify any sizeable broadening and exploit it for magnetometry, the external field must be sufficiently strong (≳1 mT), as for very weak fields, interactions between *m*_s_ = ±1 spin states due to off-axis strain hinder the observation of any changes in the ODMR spectrum.^41^

In the present work, we wanted to obtain the ODMR signals from arbitrary-oriented FND to demonstrate the magnetic sensing capabilities of very small changes δ*B* of the magnetic field, such as those created by electric currents. For this purpose, we used one wire on the described antenna board that carried currents up to ±1 A placed close to the FND-coated IFB. The current-induced magnetic fields are strongly inhomogeneous and change their intensity with distance *r* from the wire according to the Biot–Savart law.

Figure 5b depicts the acquired FF field-of-view ODMR spectra of the FND film recorded through the other end of the IFB at three positions between the antenna wires along the diameter of the IFB (double-ended dashed arrow shown in Figure 2) and three applied intensities of the current flowing through the antenna wire: 0, +1 A, and −1 A (negative currents mean a reversal of the polarity of the current source). The plots clearly show that the application of current-created δ*B* indeed shifts the edges of the broadened resonances and enables quantification of this effect for magnetometric purposes. ODMR spectra collected near the current-carrying wire are visibly broader for one current direction (*I* = −1 A), which corresponds to a higher value of the net magnetic field *B*_0_ + δ*B* than for the reverse direction (*I* = +1 A), where δ*B* opposed the bias field and decreased its net strength to *B*_0_ – δ*B*. The dependence of δ*B*(*r*) on distance *r* from the current wire is reflected in the curves depicted by various colors in Figure 5b that correspond to different spatial locations. The observed dependences become more pronounced when the signal derivatives are plotted as in Figure 5c rather than the fluorescence intensity.^37^ Consequently, the ODMR signals presented in Figure 5 illustrate that the NV centers and the IFB can be used successfully to remote sensing the magnetic field distributions.

#### 3.2.2 Demonstration of the Gradiometric Magnetic Field Sensing on Micrometer Scales

To demonstrate in more detail the feasibility of detecting spatial variations in the magnetic field generated by a current-carrying wire with an applied bias field of *B*_0_ = 3.8 mT, the microscope objective was moved transversely to the IFB and antenna wires. In this way, the distance *r* of the imaged AOI from the current wire was scanned in small steps moving away from the current wire. For each step, the microscope was refocused on the uncoated IFB facet to correct for residual unevenness of the facet’s surface. This allowed us to improve fluorescence collection and record better ODMR signals from individual AOI, and associate them with position *r*. Figure S1 shows a stitched camera image (as seen from the objective) of the entire diameter of the uncoated side of the IFB facet positioned between the MW and the current wires over the entire widths of IFB.

**Figure 6:**
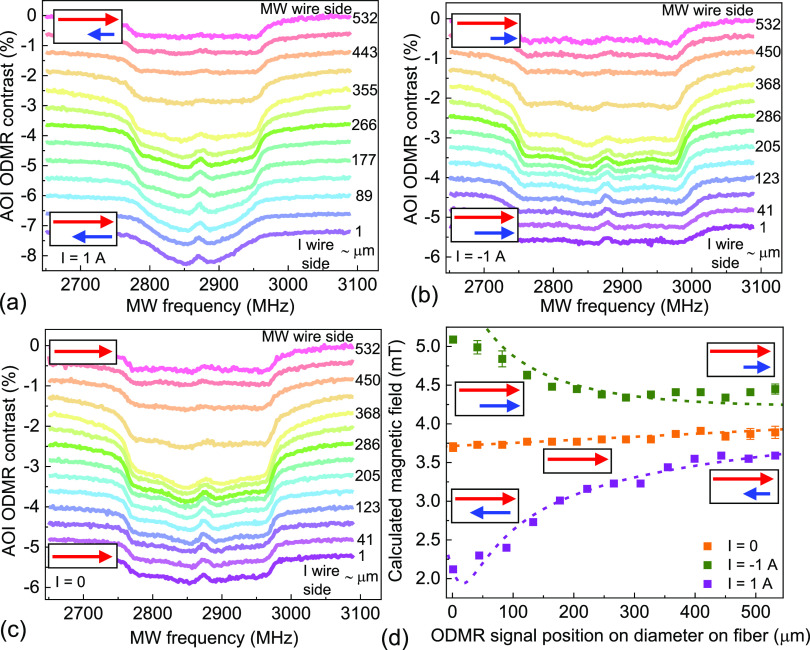
Spatial dependence of ODMR spectra caused by the magnetic field of a current-carrying wire: (a) +1 A current, (b) reversed current direction, −1 A, (c) zero current. For a zero applied current, only a fluorescence contrast value changes spatially due to the distance from the MW line, while the width of the spectrum remains constant and is set by the uniform bias field. Spectra with applied current show gradual narrowing (a) and broadening (b) as a result of vector addition of bias and created fields; (d) calculated magnetic field versus distance from the current-carrying wire. The dashed lines are the simulations for an infinite wire model. In the insets, the red arrows represent the bias magnetic field while the blue arrows show the magnitude and relative orientation of the current-generated field. The length of the arrows indicates the opposing or adding of field from current wire to bias field.

Three different current values were applied, *I* = 0, ±1 A, and ODMR spectra were acquired from AOIs. Figure 6a–c shows the resulting ODMR resonances recorded for each current value and a range of distances *r*, associated with a gradual change in magnetic field strength δ*B*(*r*), averaged over selected AOIs. For one direction of the applied current, *I* = +1 A, the ODMR peak appeared to be broadened and its contrast was reduced when the distance *r* from the current wire toward the MW wire increased, while for the reverse current direction, *I* = −1 A, the opposite behavior was observed, due to the vector addition of bias *B*_0_ and wire-created δ*B*, as marked schematically in Figure 6d. No substantial changes of spectra were observed with zero applied current; only the fluorescence contrast decreased with increasing *r* due to a weakening of the MW strength (Figure 6c). The dependences shown in Figure 6a–c enabled systematic determination of the spatial distribution of the magnetic field by analyzing the positions of external edges in the ODMR spectra.^37^

Figure 6d presents the main result of this work, the spatial dependences of the net magnetic field *B*_0_ + δ*B* (colored points) on the position between the wire for different currents, and compares them with the modeling (dashed lines) based on the Biot–Savart law. For the modeling, we took the wire dimensions: 34 μm height, 170 μm width, but instead of a real length of 2.4 mm, we extended it to infinity to simplify the modeling. The static bias field value of 3.68 mT (at *r* = 0) with an additional small gradient of 0.4 mT/mm was used. As shown, we have a very good accuracy of the two dependencies for most of the scanned distances. The accuracy of the fit is slightly reduced near the edge of the current wire (*r* = 0), due to our simplification of the infinite length of the wire. An extension of the model to include the exact dimensions of the antenna structure is fairly straightforward; however, more interesting is to note that the good match with the measurements was obtained for a specific, finite height of the NV layer in the FND film: 20 μm above the antenna plane. This finding demonstrates that the described method enables a high-resolution precision mapping of inhomogeneous magnetic fields and extending beyond a simple two-dimensional (2D) information.

The magnetic sensitivity of such mapping with FND-coated IFB is proportional to the slope (*S*) at the edge of the ODMR spectrum and inversely proportional to the square root of the photon number (*N*).^42^ The value of *N* can be obtained from a single fluorescence image, while *S* can be derived from the spectra in Figure 6. On the basis of available experimental data, we estimate that the projected magnetic sensitivity for the fiber probes discussed in this work is around 1.6 μT/√*Hz*.

## 4 Conclusions

In conclusion, we used the silanization method with intermediate heat treatments to covalently form a robust and uniform thin-film assembly of FNDs on a facet of IFB. Fluorescence wide-field microscopy images and confocal scanning maps confirm that NV fluorescence can be efficiently guided through the individual fiber cores of 2.8 μm in diameter in the IFB, allowing remote detection of optical signals. Moreover, each core conveys fluorescence information independently, i.e., from the specific FNDs that cover its surface, which allows for spatially resolved detection of NV ODMR signals.

From changes of the resonance width in the observed ODMR spectra, we retrieved information on the local magnetic field. With the FND-coated IFB facets acting as a remote sensor and IFB as an image relay, we have detected magnetic field gradients near the current-carrying structure on a scale of a few tens of micrometers. Importantly, the use of the fiber piece allowed us to move the microscope objective away from the location where the magnetic field was measured, thus removing the influence of the objective on the measurement. The improved camera system and imaging protocol allowed us to greatly enhance the signal-to-noise ratio and enabled us to reach the magnetometric sensitivity of 1.6 μT/√*Hz*. The very good agreement of the measured and simulated values demonstrated that the nanodiamond films with NV centers and image-transmitting fiber links can be successfully used in remote sensing of three-dimensional magnetic field distributions. This study enables future applications in remote magnetic field sensing and imaging, which require a high spatial resolution. We anticipate that such integrated photonic sensors can be beneficial for high-sensitivity magnetic endoscopy in bio-diagnostics and inspection of integrated electronic circuits.

## Supporting Information Available

Entire fiber facet and exact positions of the selected AOIs relative to the current and microwave wires (PDF)
