# Supplementary material for: Sensing of Magnetic-Field Gradients with Nanodiamonds on Optical Glass-Fiber Facets
Source: ACS Appl Nano Mater. 2023 Jun 14;6(13):11077–84. doi: 10.1021/acsanm.3c00887 (PMC10353531; doi:10.1021/acsanm.3c00887)
Supplement: Supplementary file 1 — an3c00887_si_001.pdf [file an3c00887_si_001.pdf]

# Supporting Information

# Sensing of Magnetic-field Gradients with Nanodiamonds on Optical Glass-Fiber Facets

Mona Jani,* Paulina Czarnecka, Zuzanna Orzechowska, Mariusz Mrózek, Wojciech Gawlik, and Adam M. Wojciechowski*

Marian Smoluchowski Institute of Physics, Jagiellonian University, Łojasiewicza, 11, 30-348 Kraków, Poland

Contact information for corresponding authors:
Mona Jani: mona.jani@uj.edu.pl
Adam M. Wojciechowski: a.wojciechowski@uj.edu.pl

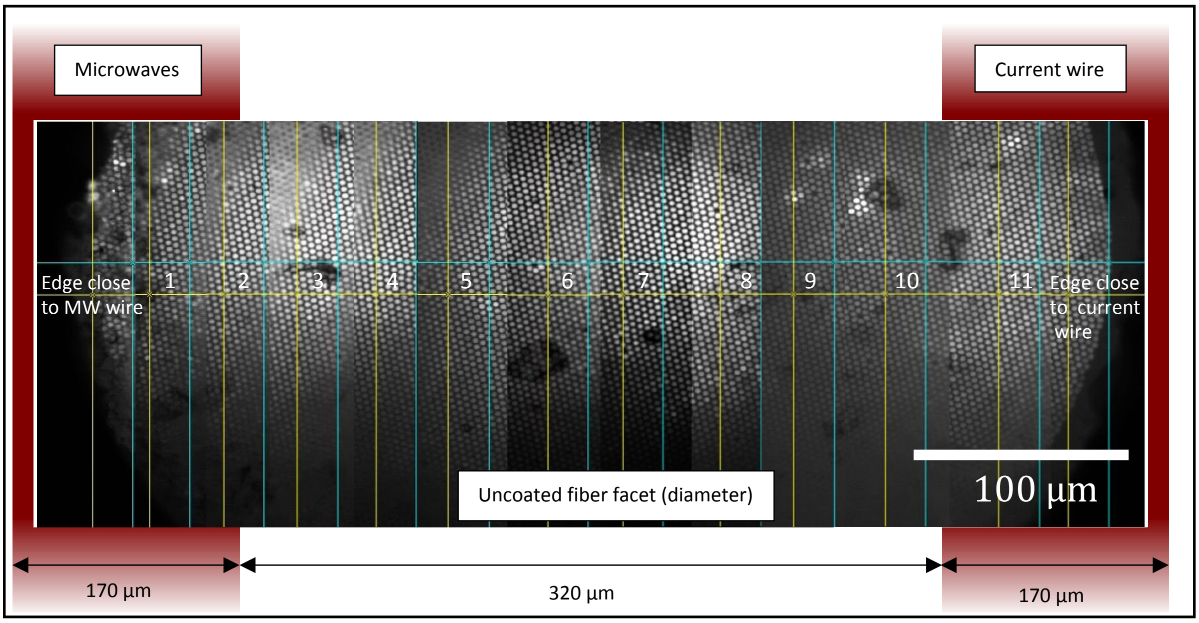


**Figure S1.** Stitched FF field view images of the entire fiber diameter were stacked to show the selected AOI in squares with numbers marked from which the ODMR signals were collected (along with showing reference MW and current wires). The blue and yellow lines are markers from software to visualize and select the area-of-interest (AOI).
